# Climate of origin affects tick (*Ixodes ricinus*) host-seeking behavior in response to temperature: implications for resilience to climate change?

**DOI:** 10.1002/ece3.1014

**Published:** 2014-03-10

**Authors:** Lucy Gilbert, Jennifer Aungier, Joseph L Tomkins

**Affiliations:** 1 James Hutton InstituteMacaulay Drive, Craigiebuckler, Aberdeen, AB15 8QH, U.K; 2 Institute of Biological and Environmental Sciences, Zoology Building, University of AberdeenTillydrone Avenue, Aberdeen, AB24 2TZ, U.K; 3 Centre for Evolutionary Biology, School of Animal Biology, University of Western AustraliaCrawley, 6009, Western Australia, Australia

**Keywords:** Adaptation, evolution, experiment, nymphs, phenotypic plasticity, questing

## Abstract

Climate warming is changing distributions and phenologies of many organisms and may also impact on vectors of disease-causing pathogens. In Europe, the tick *Ixodes ricinus* is the primary vector of medically important pathogens (e.g., *Borrelia burgdorferi* sensu lato, the causative agent of Lyme borreliosis). How might climate change affect *I. ricinus* host-seeking behavior (questing)? We hypothesize that, in order to maximize survival, *I. ricinus* have adapted their questing in response to temperature in accordance with local climates. We predicted that ticks from cooler climates quest at cooler temperatures than those from warmer climates. This would suggest that *I. ricinus* can adapt and therefore have the potential to be resilient to climate change. *I. ricinus* were collected from a cline of climates using a latitudinal gradient (northeast Scotland, North Wales, South England, and central France). Under laboratory conditions, ticks were subjected to temperature increases of 1°C per day, from 6 to 15°C. The proportion of ticks questing was recorded five times per temperature (i.e., per day). The theoretical potential to quest was then estimated for each population over the year for future climate change projections. As predicted, more ticks from cooler climates quested at lower temperatures than did ticks from warmer climates. The proportion of ticks questing was strongly associated with key climate parameters from each location. Our projections, based on temperature alone, suggested that populations could advance their activity season by a month under climate change, which has implications for exposure periods of hosts to tick-borne pathogens. Our findings suggest that *I. ricinus* have adapted their behavior in response to climate, implying some potential to adapt to climate change. Predictive models of *I. ricinus* dynamics and disease risk over continental scales would benefit from knowledge of these differences between populations.

## Introduction

Climate change is associated with shifting distributions and phenology of a broad range of species (Thomas and Lennon 1999; Bale et al. 2002; Walther et al. 2002; Parmesan and Yohe 2003; Root et al. 2003), including vectors of disease-causing pathogens, such as mosquitoes, flies, and ticks, with potential implications for infectious disease risk (Lindgren et al. 2000; Lafferty 2009; Leger et al. 2013). Vector-borne diseases are of major importance to the health and welfare of humans and other animals and to global economics (Harrus and Baneth 2005) and of particular concern is how vectors may respond to climate change, thus affecting disease risk.

**Figure 1:**
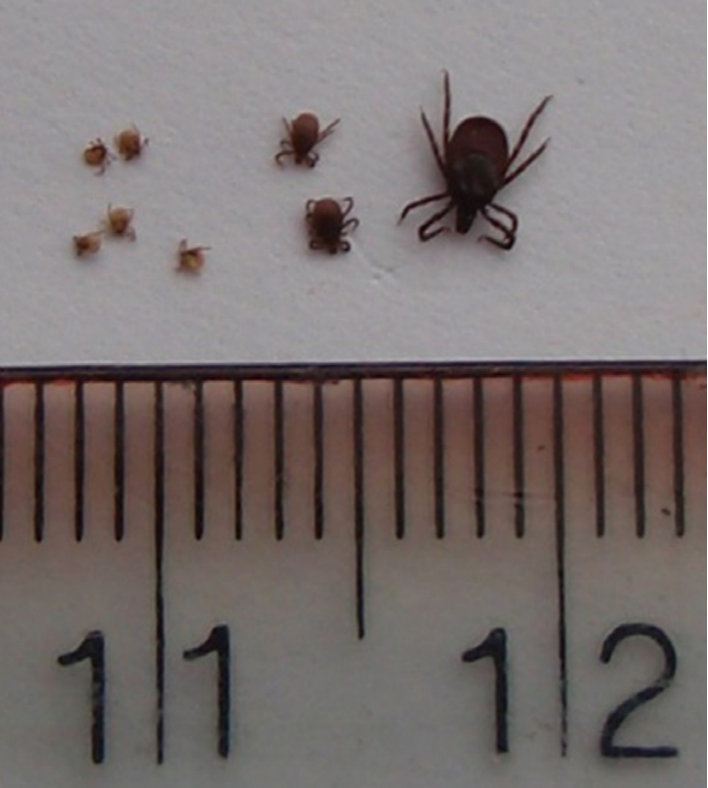
The three active life stages of *Ixodes ricinus*. From left to right: larvae, nymphs, and an adult female. Adult male not shown.

In Europe, the sheep tick, *Ixodes ricinus* (Acari: Ixodidae), is the primary vector of medically and economically important disease agents (e.g., the tick-borne encephalitis (TBE) complex of viruses, Rickettsia, Babesia, and Anaplasma species and the *Borrelia burgdorferi* sensu lato complex, the causative agents of Lyme borreliosis). *I. ricinus* has three active life stages: larva, nymph, and adult (Fig. 1), and each requires a single blood meal from a vertebrate host. In order to find a host *I. ricinus* adopts an ambush strategy, known as “questing,” that involves climbing up vegetation and waiting to grab on to a passing host. After feeding for a few days, the tick detaches from the host and develops to the next instar on the ground. As an ectotherm that spends the majority of its life cycle free-living (developing or host-seeking), *I. ricinus* is particularly sensitive to surrounding environmental conditions (Randolph 2004). Temperature and relative humidity requirements for *I. ricinus* questing, development and survival are thought to be the principal factors limiting the geographic range of this species (Lindgren et al. 2000; Gray et al. 2009). Recent expansion of *I. ricinus*' European range to higher latitudes and altitudes, increases in its population density (Lindgren et al. 2000; Danielová et al. 2006), and corresponding increases in reported cases of tick-borne diseases (Lindgren 1998) have been attributed partly to climate warming, among other factors (reviewed by Medlock et al. 2013; Leger et al. 2013).

Questing is a behavior essential to each instar of *I. ricinus* if it is to feed (and therefore survive and molt to the next instar, or lay eggs) and is also the behavior that puts hosts at risk of parasitization and pathogen infection. Understanding how temperature affects questing is therefore central to underpinning the potential impact of climate warming on *I. ricinus*. Field surveys have estimated a weekly average maximum daily air temperature of approximately 7–8°C to be necessary for the onset of questing of *I. ricinus* nymphs following winter diapause (a state of behavioral inactivity; Randolph 2004) in *I. ricinus* populations in central Scotland (MacLeod 1935), Switzerland (Perret et al. 2000), and northern Italy (Tagliapietra et al. 2011). Assuming phenotypic variation between individuals (so that not all ticks start questing at exactly the same temperature) we can predict that, as temperatures increase, the proportion of the tick population questing should increase. This should occur until the increasing saturation deficit (a measure of “drying power” of the air that also increases with temperature for a given relative humidity; Perret et al. 2000) forces ticks to descend to the moist litter-layer to re-absorb water to prevent desiccation-induced mortality (Perret et al. 2004), although such warm and dry conditions are unlikely during the early period of the tick questing season (Tagliapietra et al. 2011).

Various arthropod species that occupy wide latitudinal and altitudinal ranges show geographic variation in the thermal conditions they require for certain behaviors (Ayrinhac et al. 2004; Castañeda et al. 2005). Adaptation to local conditions allows species to exist over wide geographic ranges and aids persistence (i.e., resilience) in response to changing environmental conditions. *Ixodes ricinus* occupies a large geographic area (ranging latitudinally from North Africa to Scandinavia, and longitudinally from Ireland to Russia; for example, Randolph et al. 2002) over which climatic conditions vary considerably. To predict how resilient *I. ricinus* might be to climate change and to allow the application of predictive models of tick population dynamics in relation to climate over wide geographic areas, we need to determine the geographic variation in the temperature-dependent questing behavior of *I. ricinus*. We hypothesize that *I. ricinus* populations will have adapted their questing response to temperature in accordance with the local thermal climate, in order to maximize their survival. If this hypothesis is supported, it suggests that *I. ricinus* can adapt and therefore may have the potential to be resilient to climate change, given enough time to adapt to the new climate.

Here, we quantify differences in questing behavior in *I. ricinus* nymphs from four populations across a climatic gradient in response to experimentally increased temperatures. If our hypothesis that *I. ricinus* have adapted their questing behavior to local thermal climates is correct, we predict that a higher proportion of the tick population will quest at lower temperatures if they are from cooler rather than warmer climates. In this study, we focus on questing behavior at the lower part of the temperature range for questing, because these temperatures are most relevant to the start of questing in spring, thereby altering phenology and the length of the tick questing season.

## Materials and Methods

### Tick collection

**Figure 2:**
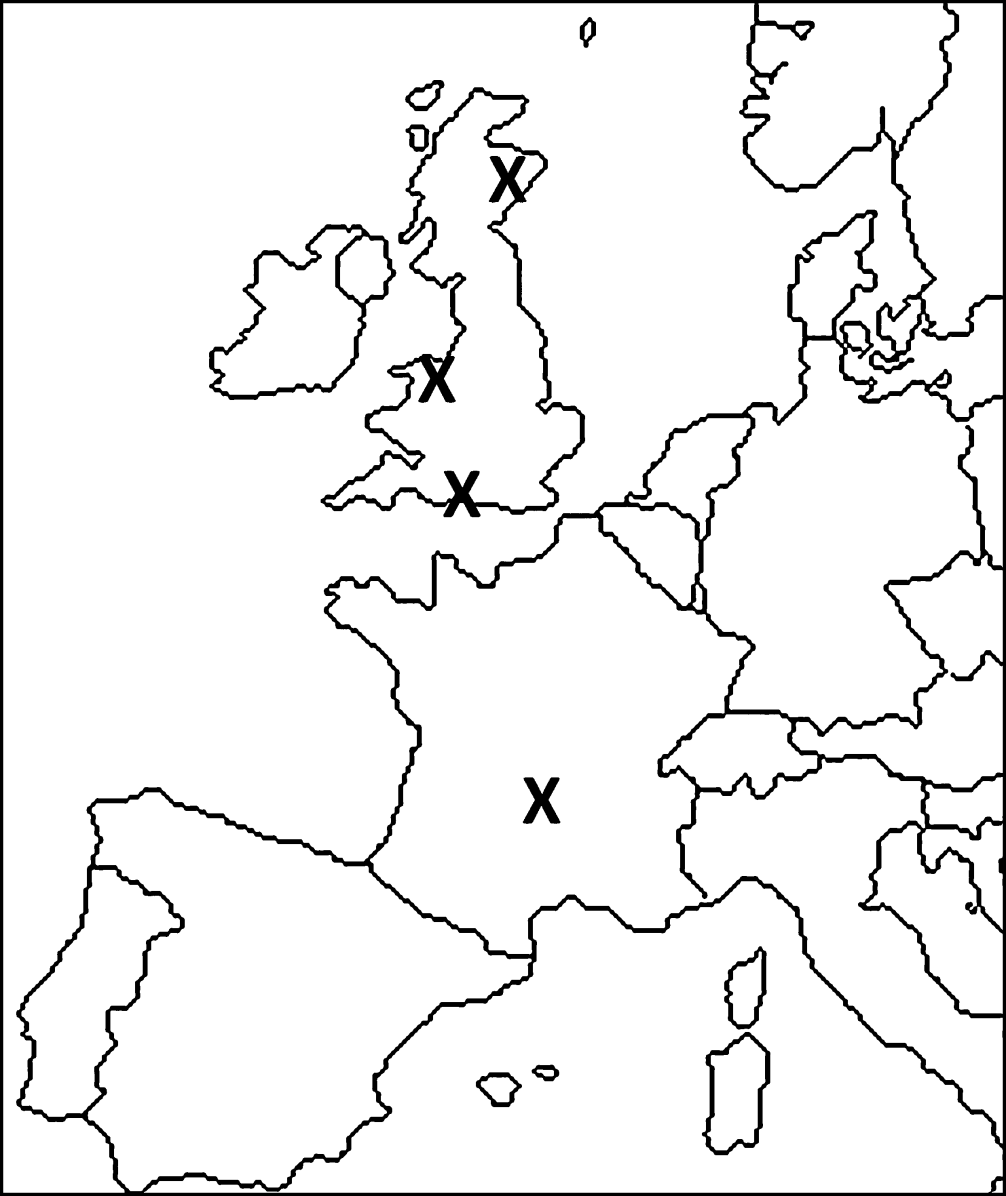
Map of western Europe showing the locations of each site from which ticks were collected for the experiments. From top to bottom (North to South): Aberdeenshire, northeast Scotland; Denbighshire, North Wales; Hampshire, South England; Auvergne, central France.

**Figure 3:**
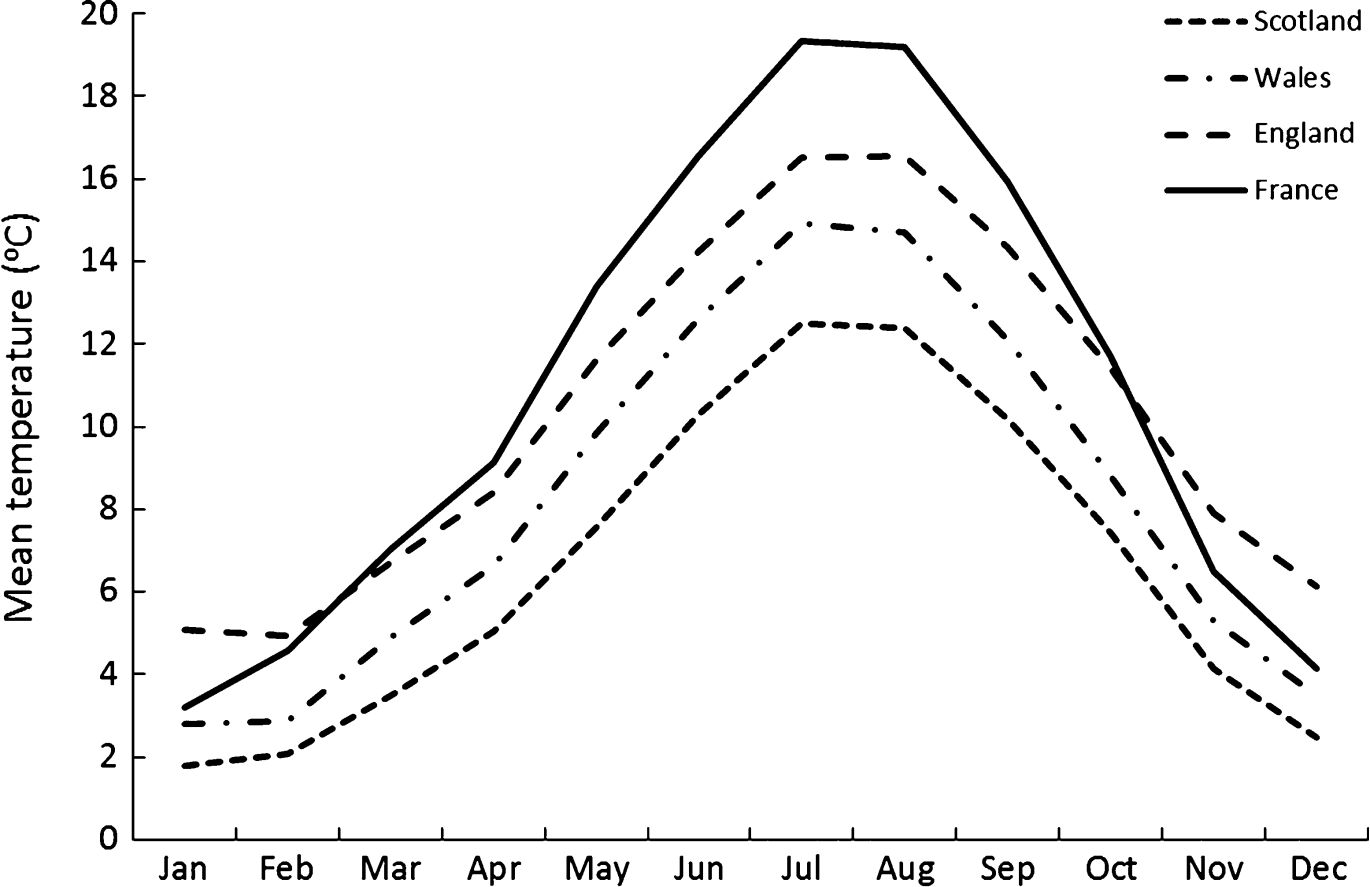
Estimated mean monthly temperatures (1971–2000) at the four geographic locations (northeast Scotland, North Wales, South England, and central France) from which ticks were sampled. Data were obtained from the UK Meteorological Office (Meteorological Office 2014) and from Meteo France via the World Weather Information Service (2014) for the nearest weather station to each location and then estimated for each tick sampling location by adjust for altitude.

*Ixodes ricinus* were collected from four geographically distinct locations: northeast Scotland, North Wales, South England, and central France (Table 1). Sites were chosen to represent a cline of thermal climates (Fig. 2); we used a latitudinal gradient to achieve this (Fig. 3). Within the latitude remit, sites were chosen primarily on the basis that we had prior knowledge that they all had high tick densities that enabled the collection of hundreds of ticks within a few hours. This was essential to minimize the potentially confounding factor of different tick storage periods both between and within sites. However, this meant that some of the sites differed in habitat. Different habitats can create different micro-climates, for example, tall and dense vegetation that creates a canopy can modify the climate slightly, by buffering ticks from the extremes of heat and wind; in contrast, open habitats with little ground vegetation, such as short cropped pastures are more exposed to the wind and direct sunshine. While some of our sites differed in habitat (Table 1), all had a type of vegetation that similarly created good canopy cover (trees, bracken, or heather). We are therefore confident that the ticks from each site were subject to the intended thermal climate gradient, from the coldest in northeast Scotland to the warmest climate in central France.

**Table 1:** Details of the four geographic locations from which ticks were collected.

Region	Area	Latitude, longitude	Altitude (m)	Estimated yearly mean max. temp. (°C)	Vegetation type	Main large tick hosts present
Northeast Scotland	Aberdeenshire	56°54′N, −2°31′E	300	9.9	*Calluna*-dominated heath	Red deer *Cervus elaphus*
North Wales	Denbighshire	53°5′N, −3°15′E	230	12.4	Bracken-dominated rough pasture	Sheep *Ovis aries*
South England	Hampshire	50°51′N, −1°38′E	40	13.8	Mixed deciduous woodland: *Quercus, Fagus, Pinus*	Roe deer *Capreolus capreolus*, fallow deer *Dama dama*
Central France	Auvergne	45°47′N, 03°27′E	380	16.0	Mixed deciduous woodland: *Quercus, Fagus, Acer*	Roe deer *Capreolus capreolus*

To control for interstadial differences in temperature thresholds (Clark 1995; Randolph 2004) only nymphs were used in our experiments. As a tick's fat reserves diminish through the questing season, potentially affecting activity levels (Crooks and Randolph 2006; Herrmann and Gern 2012), nymphs from all sites were collected within the last 2 weeks of May 2012. However, ticks from warmer climates are likely to start questing earlier in the season (and therefore have used up slightly more fat) than cooler climate ticks. To mitigate this to some extent, ticks from central France were collected first, followed 10 days later by the English, then Welsh, and lastly the Scottish ticks (which were collected 2 weeks after the French ticks). Nymphs were collected by dragging a 1 m × 1 m piece of blanket material along the ground. Conditions during tick collection over all sites ranged from 17 to 22°C and 60–70% relative humidity (recorded using Tinytag digital data loggers placed on the ground vegetation). Nymphs were stored in plastic boxes (5 cm × 10 cm), containing damp tissue to maintain high humidity, and refrigerated at 4–6°C for 1–4 days until the experiments began.

### Tick questing in response to increased temperature

Nymphs from each of the four populations were put in tubes made from nylon mesh (250 *μ*m diameter) that were 3 cm diameter, 13 cm high and glued at the base to a 3 cm diameter Petri dish. Each tube was filled with 1.5 cm of wet sand, then 1 cm of damp moss, to provide a moist environment where ticks could rehydrate if necessary as they would in nature. Immediately after adding the ticks, the tops of the tubes were sealed. Twelve tubes, each with 30 nymphs (i.e., 360 nymphs in total per site), were used for each population, except for the North Wales population that had 10 tubes (i.e., 300 nymphs in total) due to fewer ticks collected from that site.

The mesh tubes containing ticks from the UK sites were allocated equally between two portable incubators (Memmert IPP 200; Memmert, Schwabach, Germany). In order to control for any potential variation in conditions between the two incubators, an equal proportion of tubes from each population were allocated to each incubator, and the temperature and relative humidity in each incubator was calibrated by Tinytag digital data loggers. In addition, tubes from each population were arranged alternately in sequence to control for any potential effects of position within the incubator. For the French ticks, in order to ensure identical experimental conditions to the UK ticks, the same individual incubators were used, the temperature and relative humidity were calibrated with the same Tinytag digital data loggers and the same human observers were used for all populations. Experiments on the French ticks took place in France 2 weeks prior to the UK experiments. This was necessary, first, to ensure that ticks from all populations were subject to similar conditions for a similar length of time between collection and conducting the experiment (otherwise, the French ticks would have been in storage for a week longer than the UK ticks) and, second, to avoid the biosecurity risk associated with transporting live specimens across country borders.

In nature, a tick's response to increasing temperature may be confounded by variations in saturation deficit depending on the relative humidity at the time (which depends on the location, vegetation, and recent precipitation). Therefore, to ensure that we were testing the effect of temperature per se on questing we maintained the incubators at >90% humidity (as confirmed by Tinytag digital data loggers) by placing damp tissue around the interior. This also promoted maximum tick survival (MacLeod 1935) to minimize any error in our counts due to mortality over the experimental period.

To control for possible effects of photoperiod on questing behavior (Lees and Milne 1951; Randolph 2004), a “summer-time” regime of 16-h light: 8-h dark was maintained. Incubators were initially set to 5°C for 2 h, to ensure that no ticks were questing before the start of the experiment. The temperature of the incubator was then raised to 6°C for 24 h, marking the first experimental temperature and then further increased by 1°C every 24 h. At each temperature, we counted the number of ticks questing in each tube five times, at 06:30, 09:00, 13:30, 18:00, and 22:30 h, and immediately after the count at 22:30, we increased the temperature of the incubator by 1°C. Thus, 8 h had lapsed between setting the new temperature and the next count at 06:30. By allowing 24 h and five counts for each temperature, we could gauge whether or not the proportion of ticks questing stabilized at each temperature (see Supporting Information). Experiments on the UK populations ran for 10 days, covering a temperature range of 6–15°C. Due to time restrictions, experiments on the French ticks ran for 8 days, covering 7–14°C. Therefore, there were a total of 50 observations for each tube of UK ticks over the 10-day experimental period and 40 observations for each tube of French ticks over the 8-day experimental period. We had to ensure minimum tick mortality over the experiment by keeping the experimental period as short as possible, so there was a trade-off between the period at each temperature and the number of temperatures tested. For example, if we had allowed more than 24 h for each temperature, we would either have needed a longer experimental period (and therefore higher tick mortality), or not covered the critical range of temperatures. Previous trials (unpublished data) enabled us to conclude that the best compromise was an experimental period of no more than 10 days with no less than 24 h for each temperature. A temperature range of 6–15°C was chosen to encompass as much of the natural range at the sites as possible and, importantly, to approximately reflect the temperature range likely to be experienced by *I. ricinus* in spring as questing increases (this study may be less relevant to the end of the questing season because behavioral diapause in late summer or autumn is thought to be driven by reduction in day length as well as temperature; Randolph et al. 2002). Figure 3 shows that our chosen temperature range of 6–15°C achieves this by encompassing the mean monthly air temperatures estimated from the end of April to the end of October for the site in northeast Scotland (the entire tick activity season for this area; Ruiz-Fons and Gilbert 2010); the end of March to mid-November for North Wales, and the start of March to the end of November, but not including July, August or the first half of September for South England (encompassing the period when tick questing increases for North Wales and South England; Randolph 2004). For central France, our chosen experimental temperature range of 7–14°C encompasses early March to the start of December, but not including June, July, August or September. Our results confirmed that our chosen temperature range covered most of the questing range of *I. ricinus* nymphs over all from the four populations (from 5% to 91% ticks quested between 5°C and 15°C).

In order to minimize stimulation of questing in response to the observer (e.g., CO_2_, vibrations and shadows), incubators were located in a quiet laboratory and their internal glass doors were kept closed during observations. Observers moved slowly and quietly during counts.

The Tinytag digital data loggers were used not only to calibrate the temperature settings on each incubator but also subsequently to log the temperature and relative humidity inside the incubators every 5 min. These data showed that humidity was maintained at >90% and the incubators took approximately 10 min to stabilize from one temperature to the next. They also confirmed that all tubes were subject to the same temperatures and relative humidity (including, crucially, the French ticks that were trialed in the same incubator but a week earlier).

At the end of each experiment, all tubes were dismantled and the numbers of live and dead ticks counted.

### Climate data

To estimate the local thermal climatic conditions typically experienced by the four tick populations in their natural environments, 1971–2000 long-term average climate data were obtained from the UK Meteorological Office (Meteorological Office 2014) and from Meteo France, via the World Weather Information Service (2014). Data came from the nearest weather station to each location as follows: Craibstone, 102-m above sea level (m asl), 35-km NNE of the northeast Scotland collection site; Shawbury, 72 m asl, 50 km southeast of the North Wales collection site; Everton, 16 m asl, 13 km South of the South England collection site; Clermont Ferrand, 331 m asl, 25 km E of the French collection site. The monthly and annual mean temperatures at each tick sampling site (Table 1; Fig. 3) were estimated by adjusting the data from these weather stations to account for differences in altitude by subtracting 6.4°C for every 1000-m elevation gain, which is the lapse rate under normal atmospheric conditions (Moore 1956).

It was important to confirm that the temperatures we used in the experiments coincided with the temperatures associated with ticks naturally increasing their questing at each site. Therefore, for each site, we used our results to predict which months had temperatures associated with an increase in questing from 20% to 80%, and compared this with our experimental temperatures. This also helped us assess how robust our questing season estimates were for each site.

### Exploring the impact of climate change

To estimate how climate change may affect future temperatures experienced by each tick population, we adjusted the estimated 1971–2000 mean monthly temperatures (estimated as described above for each sampling location) in accordance with the IPCC temperature change projections for each region we sampled (Christensen et al. 2007). We used the IPCC-projected temperature changes 1980–1999 and 2080–2099, averaged over 21 models (Christensen et al. 2007). For northeast Scotland and North Wales, these temperature change projections are +2.5°C in winter (December, January, February) and +2°C in summer (June, July, August; Christensen et al. 2007). We therefore assumed the midpoint of +2.25°C for spring and autumn (March, April, May, September, October, and November). For South England, these temperature change projections are +2.5°C in both winter and summer (Christensen et al. 2007) and we assumed also for spring and autumn. For central France, these temperature change projections are +2.5°C in winter and +4°C in summer (Christensen et al. 2007). We therefore assumed the midpoint of +3.25°C for spring and autumn.

We then explored the estimated impact of climate change on the proportion of ticks questing by applying the results of our questing experiments to the projected changes in monthly mean temperatures at each sampling location. For this, we graphically compared the estimated proportion of *I. ricinus* nymphs questing for estimates of the 1971–2000 monthly mean temperatures with those questing for our estimates of the temperatures in 2080–2099 from the IPCC temperature change projections as described above (Christensen et al. 2007). For months when the temperatures tend to be colder or warmer than our experimental temperatures, our estimated proportions of nymphs questing during those months had to be estimated by extrapolation and are therefore less robust.

### Data analysis

To account for nymphs found dead in each tube at the end of the experiment, and in the absence of information on when each tick died, we assumed a constant death rate over the course of the experiment (after Van Es et al. 1999). Tick questing activity was then calculated at each observation as a proportion, that is, the number of ticks observed questing divided by the estimated total number alive inside the tube at that time.

A generalized linear mixed model was used to analyze the proportion of ticks in each tube that were questing, using the GLIMMIX procedure in SAS version 9.1.3 (SAS Institute 2002). This procedure was chosen in order to allow for the binomial nature of the response variable (proportion of ticks questing) as well as the presence of random effects. The model included tick population (i.e., site name), temperature, temperature^2^, and their interactions (tick population*temperature, tick population*temperature^2^) as fixed effects, and random effects were “block” (representing the two incubators used simultaneously for the UK populations plus the separate trial (albeit using the same incubator) for the French population) and tube (as 50 count observations were made for each tube for each population). The quadratic term, temperature^2^, was included in the model because there was a clear curved relationship between temperature and the proportion of ticks questing. Type 1 tests of the fixed effects showed temperature^2^ explained more variation than did temperature; therefore, final models included temperature^2^ rather than temperature per se.

Generalized linear mixed models were then run for each temperature (6–15°C) individually with proportion of questing as the response variable, tick population as the fixed effect and block and tube entered as random effects. This enabled us to identify the temperatures at which the proportion of ticks questing varied significantly between populations. Post hoc analyses, using Tukey–Kramer comparisons of the least squares means, were performed to indicate the populations between which there was significant variation in the proportion of ticks questing at each temperature.

Although tick populations were collected from climates along a thermal climate gradient due to differences in latitude, each site differed in other ways, such as rainfall, snow cover, habitat, and hosts. For this reason, and to explore the nature of any relationship between questing and thermal climate, we reran these generalized linear mixed models, but used climate parameters as continuous variables as fixed effects in place of site name (a categorical variable). Climate parameters used were mean air temperature in May (because our results suggested that this month experienced temperatures associated with the steepest increase in nymph questing) and the number of weeks per year with a mean temperature of at least 10°C (because our results suggested that most ticks quested above 10°C in all populations). These climate parameters were entered separately and sequentially in separate models. For these models, we used questing data from 9°C, because the results of our experiments indicated this temperature to be closest to the temperature at which 50% of nymphs quested for all populations (8.1°C, 8.5°C, 9.1°C, and 10.2°C for Scottish, Welsh, English, and French nymphs, respectively).

## Results

Mortality rates of *I. ricinus* nymphs in the mesh tubes during the experiments were low, varying from 0 to 5 (mean 0.08) ticks found dead of the 30 initially added to each mesh tube. The average mortality was 0.83%, 0.68%, 3.39%, and 4.81% for Scottish, Welsh, English, and French ticks, respectively. The following results have taken into account the mortality in each individual tube assuming a constant linear mortality rate over the course of each experiment.

### Tick questing in response to increased temperature

**Figure 4:**
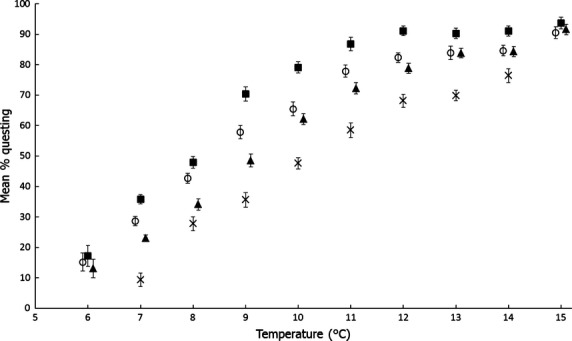
The effect of temperature on the proportion of ticks questing in *Ixodes ricinus* sampled from northeast Scotland (black squares), North Wales (open circles), South England (black triangles) and crosses (central France). French ticks were not tested at 6°C or 15°C. Unadjusted means with standard error bars are shown. Points for South England and North Wales have been shifted slightly to allow room for standard error bars.

Some (17.2%, 15.2%, and 13% for Scottish, Welsh, and English ticks, respectively) ticks in all the UK tick populations were already questing at 6°C, the initial temperature at which observations were started, and 9.4% of French ticks were questing at their initial temperature of 7°C (Fig. 4). Almost all (93.7%, 90.5%, and 91.6% for Scottish, Welsh, and English ticks, respectively) of the UK ticks were questing by the time the final temperature (15°C) was reached, and 76.4% of French ticks were questing at their final temperature of 14°C (Fig. 4).

Allowing 8 h between increasing the temperature each night and counting the ticks the next morning appeared to be enough time for the ticks to respond to each temperature: there was a clear step-change in the proportion of nymphs questing between temperatures compared with the five within-temperature counts (Fig. S1). However, for the first temperature of the experiments (6°C for UK ticks, 7°C for French ticks), there was an obvious increase in questing over at least the first three counts; this was after the ticks had been kept at 5°C to prevent questing. Such an increase in questing over the five counts may also be occurring for additional temperatures for the French ticks (Fig. S1). Perhaps, therefore, warm climate ticks may take longer to respond to temperature increases than cool climate ticks.

The overall proportion of ticks questing increased significantly with temperature^2^ (*F*_1,2126_ = 670.91, *P* < 0.0001) and differed between populations (*F*_3,2126_ = 10.60, *P* < 0.0001). There was a highly significant interaction between population and temperature^2^ (*F*_3,2126_ = 15.34, *P* < 0.0001) indicating that the pattern of ticks questing with temperature varied between populations. Within the range of temperatures tested the proportion of ticks questing was generally lowest in the central France population, followed by the South England population, then the North Wales population, with the highest proportion of ticks questing in the northeast Scotland population; these differences were greatest at low and intermediate temperatures and negligible at the higher temperatures tested (Fig. 4).

To explore quantitatively the nature of the significant interaction between population and temperature, we analyzed each individual temperature separately and found significant differences between populations in the proportion of ticks questing at all temperatures except the minimum (6°C) and maximum (15°C) tested (note that French ticks were not tested at these temperatures; Table 2). The greatest population differences in the proportion of ticks questing occurred at the lower temperatures tested (7–10°C; excluding 6°C, at which the French population was not tested; Fig. 4 and Table 2).

**Table 2:** Results of generalized linear mixed models run for each temperature separately to test in which temperatures the percentage of ticks questing varied between populations. The French ticks were not tested at 6°C or 15°C. Df (num, den) = numerator and denominator degrees of freedom.

Temperature (°C)	Df (num, den)	*F*-value	*P*-value
6	2,136	1.63	0.1995
7	3,184	17.77	<0.0001
8	3,184	7.01	0.0002
9	3,184	8.18	<0.0001
10	3,184	8.62	<0.0001
11	3,184	6.84	0.0002
12	3,184	6.48	0.0003
13	3,184	4.30	0.0059
14	3,184	3.18	0.0253
15	2,136	0.82	0.4431

The proportion of ticks questing differed most between *I. ricinus* populations from northeast Scotland and central France, closely followed by the comparison between northeast Scotland and South England populations, while the South England and North Wales *I. ricinus* populations did not differ significantly in the proportion of ticks questing for any of the temperatures tested (Table 3).

**Table 3:** The temperatures at which significant differences (*P* ≤ 0.05) in the percentage of ticks questing were found between pairs of populations, as indicated by Tukey-Kramer post hoc analysis. The French ticks were not tested at 6°C and 15°C.

Populations	Temp (°C)	DF	*t*-value	*P*-value
France vs. South England	7	184	4.13	0.0003
France vs. North Wales	7	184	−5.47	<0.0001
	8	184	−3.05	0.0138
France vs. northeast Scotland	7	184	−6.96	<0.0001
	8	184	−4.23	0.0002
	9	184	−3.67	0.0018
	10	184	−4.19	0.0003
	11	184	−3.03	0.0147
	12	184	−2.97	0.0174
	13	184	−2.91	0.0208
	14	184	−2.66	0.0423
South England vs. North Wales	No significant differences			
South England vs. northeast Scotland	7	184	−2.97	0.0235
	8	184	−2.88	0.0227
	9	184	−4.21	0.0002
	10	184	−3.98	0.0006
	11	184	−3.97	0.0006
	12	184	−3.82	0.0010
	13	184	−2.61	0.0482
North Wales vs. northeast Scotland	10	184	3.08	0.0125
	11	184	2.79	0.0295

### Tick questing by climate gradient

**Figure 5:**
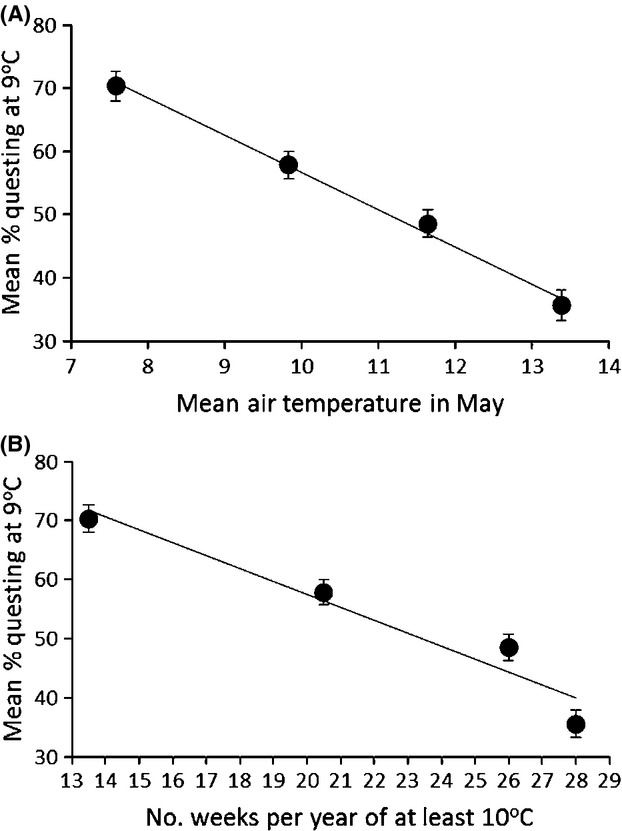
The association between the proportion of nymphs questing at 9°C from the experiments and (A) mean temperature in May and (B) the number of weeks per year of at least 10°C. The line of best fit, unadjusted means, and standard error bars are shown.

There was a highly significant negative association between the proportion of nymphs questing at 9°C and the mean air temperature in May (*F*_1,183_ = 28.55, *P* < 0.0001) and the number of weeks per year of at least 10°C (*F*_1,184_ = 24.18, *P* < 0.0001) at the site from which ticks were collected (Fig. 5).

### Exploring the impact of climate change

**Figure 6:**
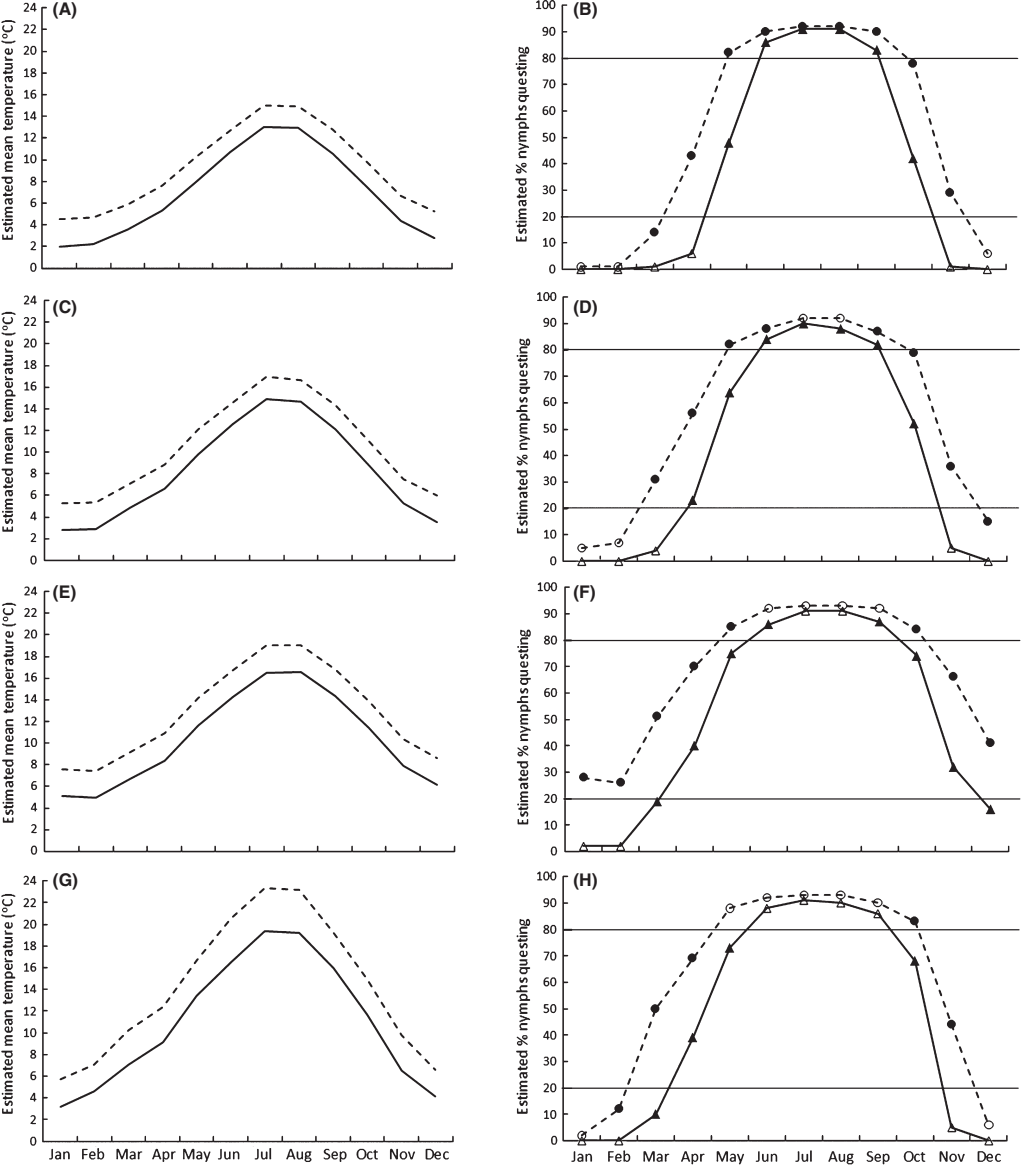
Estimated mean monthly temperatures (left hand side) and projected mean percentage of active nymphs questing (right hand side) for current (1970–2000; solid lines) and future (2080–2099; dashed lines) temperature estimates for each tick sampling site (A and B: northeast Scotland; C and D: North Wales; E and F: South England; G and H: central France). See text for details of temperature estimation methods. Filled (black) symbols indicate the more robust estimates of the percentage of active nymphs questing because the temperatures were used in the experiment; unfilled (white) symbols indicate values estimated by extrapolation because they relate to temperatures not used in the experiment. Horizontal lines are placed where 20% and 80% nymphs are predicted to be questing as a visual aid.

Figure 6 shows the difference in estimated mean monthly temperatures between 1970–2000 estimates and 2080–2099 estimates for each tick sampling location. Figure 6 also shows our projected estimates of the proportion of active nymphs questing for each month for 1970–2000 temperature estimates and 2080–2099 temperature estimates. Note that these are not estimates of the total numbers of questing nymphs for each month, nor proportions of the whole nymph population, nor the probability of questing; instead, these values are the estimated mean proportion of nymphs questing out of the active nymph population that are still available and able to quest at that time (i.e., nymphs that are feeding or have fed and are therefore molting, developing or in behavioral diapause, or nymphs with fat exhaustion are not included in the calculation).

1. *Comparing the experimental temperatures to local seasonal temperatures:* It was important to confirm that the temperatures we used in the experiments were relevant to the time of year when ticks were increasing their questing in nature. Figure 6 shows that, for the 1970–2000 estimated monthly temperatures, the experimental temperatures of 6–15°C encompassed most, if not all, of the months during which 20% or more nymphs were predicted to be questing for northeast Scotland and North Wales. For South England, our experimental temperatures did not reach as high as July and August temperatures, and for central France, our experimental temperatures (7–14°C) did not reach as high as June, July, August, and September temperatures. Therefore, our estimated proportions of active nymphs that are questing (i.e., discounting those feeding, developing, molting or in diapause) during those months are extrapolations and less robust. However, importantly, the main period of increase in questing (when the proportion of active nymphs questing increased from 20% to 80%) was covered by our experimental temperatures (Fig. 6). For the estimated 2080–2099 temperature projections, our experimental temperatures are still within the main questing months (20% or more nymphs predicted to be questing) for northeast Scotland, but questing estimates are less robust during July and August for North Wales, June–September for South England and May–September for central France (Fig. 6).
2. *Theoretical estimated length of the questing season:* Figure 6 helps us to predict that, for 1970–2000 estimated monthly temperatures, an average of more than 20% of active nymphs quest at any one time over a 6 months period in northeast Scotland, over 7 months in North Wales, over almost 9 months in South England and nearly 8 months in central France. An average of at least 80% of active nymphs were projected to quest at any one time over 3 months in both northeast Scotland and North Wales and over 4 months in both South England and central France. Under the climate change scenario (i.e., 2080–2099 estimated monthly temperatures), we projected a potential expansion by approximately 1 month at both the start and end of the questing season (i.e., an increase of 2 months) for ticks in northeast Scotland, North Wales, and central France (whether measured using 20% or 80% nymphs questing). Ticks in South England were projected to always experience temperatures where at least 20% of the active nymph population could theoretically be questing if they were available, active, and able to do so (i.e., not feeding, developing, molting or in diapause or with fat exhaustion).

## Discussion

This study tested the hypothesis that *I. ricinus* show adaptation in their questing behavior to local thermal climates, by testing for differences between *I. ricinus* nymphs from a thermal gradient (achieved here using a latitudinal cline) in their questing response to increasing temperatures. As predicted, we found that, for the range of temperatures tested on all populations (7–14°C), a higher proportion of *I. ricinus* nymphs quested if they were from cooler climates (such as northeast Scotland) compared with warmer climates (such as central France), and this difference was greatest at the lower-medium temperatures (7–11°C). This has implications for their potential resilience to climate change.

### Population differences in questing response to temperature

The proportion of ticks questing increased dramatically as temperatures were raised above 6°C shows that, as found by several previous studies (e.g., MacLeod 1935; Clark 1995), cold temperatures inhibit questing in *I. ricinus*. We found that 13–17% of nymphs from the three UK populations were already questing at 6°C, and 9% of French nymphs were questing at 7°C. By 14°C, questing had increased to 76–90%. These proportions questing depended on the population, and our most important finding was that a higher proportion of *I. ricinus* nymphs quested at cooler temperatures if they were from cooler climates, whereas very few quested at cooler temperatures if they were from warmer climates, as we predicted from our hypothesis that *I. ricinus* have adapted to their local thermal climates. We demonstrated this first by showing simply that populations differed in the proportion of ticks questing in the predicted way (i.e., a higher proportion of Scottish ticks quested at cool temperatures than did Welsh and English ticks, with the lowest proportion questing being from central France). However, this analysis could not elucidate the nature of the relationship between questing and thermal climate per se, and furthermore, these four sites also differ in factors other than temperature, such as rainfall, habitat, hosts and, almost certainly, prevalence in ticks of *B. burgdorferi* sensu lato that can affect tick activity (Herrmann and Gern 2010, 2012). Therefore, we also related tick questing to quantitative climate variables, rather than merely geographic location, and found strong negative linear relationships between the proportion of ticks questing at a given cool temperature (in this case 9°C) and thermal climate.

What are the potential mechanisms for these observed differences between *I. ricinus* populations in questing at cool temperatures? Natural selection may have changed the frequency of genotypes such that the response to temperature may have a genetic basis. Alternatively, or in addition, the differences between populations could be a result of phenotypic plasticity, whereby ticks from all populations may have the ability to quest at low temperatures, but these genes are expressed only in the appropriate climate. Phenotypic plasticity is the most important factor explaining the geographic variation in thermal tolerances of both *Drosophila melanogaster* (Ayrinhac et al. 2004; Hoffmann et al. 2005), and the tsetse fly, *Glossina pallidipes* (Terblanche et al. 2006), and may be instrumental in determining resilience to climate change for certain species (Molina-Montenegro and Naya 2012). For phenotypic plasticity to explain our results, the nymphs we used must have experienced an environmental trigger, such as their local climate when they were larvae or eggs or via maternal effects, whereby the environment experienced by the mother affects the phenotype of the offspring. Further research would be required to identify which mechanism contributes most to the population differences we found.

If ticks from cool climates quest at lower temperatures, why don't ticks from warm climates also quest at equally lower temperatures? The purpose of questing is to find a host, essential in gaining the crucial blood meal to enable survival to the next life stage. Questing and finding a host early in the season may benefit all ticks, so they can complete their life cycle more quickly. However, given our findings that cool climate ticks quest at cooler temperatures than do warm climate ticks, presumably, for warm climate ticks (but not cool climate ticks), the benefits of questing at low temperatures are not enough to outweigh the costs. The costs of questing include risks of desiccation, predation and spending valuable energy (when a tick uses up its fat resources, it will die; Randolph et al. 2002). Therefore, the questing period is finite, depending on fat resources, before the tick dies (if it does not gain a blood meal from a host). One putative hypothesis to explain stronger selection on cold than on warm climate ticks to quest at cold temperatures is as follows. Suppose that, for example, (1) most nymphs need temperatures of at least 10°C to quest (in this study, 40–80% of ticks quested at 10°C depending on population) and (2) they can survive for 4 months above this temperature before using up their energy resources and dying (Steele and Randolph 1985). A warm climate might have 6 months of the year of at least 10°C (e.g., central France in this study), that is, warm enough to quest, in which case, there is little selective advantage for a tick to extend this season by questing at colder temperatures. However, a cold climate might have only 3 months of the year of at least 10°C (e.g., northeast Scotland in this study), providing a selective advantage to ticks that quest at cooler temperatures (so they can quest for longer, thus increasing the probability of finding a host). This theory would need further experiments to test empirically.

### Impacts of climate change

We used our empirical data from the experiments to project theoretical proportions of active ticks that may quest under climate change. From this, the projections estimated that, under a climate change scenario for projected 2080–2099 temperature increases (Christensen et al. 2007), the questing season for active ticks might increase by a month in spring for all populations tested and at least 20% of active nymphs would be able to quest (theoretically, according to temperature alone) at any one time over the whole year in South England. It is important to emphasize that our projections are for the proportion of active ticks (those that are not feeding, developing, molting, or in diapause) that are questing, which is different from the absolute number of ticks questing. This difference is especially great in autumn when many ticks will have already fed or entered behavioral or morphogenetic diapause and are therefore no longer active until the following year (Randolph et al. 2002). Indeed, cessation of questing in autumn is likely to be driven by shortening day length as well as temperature (Randolph et al. 2002). This means that both our estimated projections and our empirical results are more applicable to spring and early summer rather than to autumn.

Our findings strongly imply that *I. ricinus* ticks have adapted to their local thermal climates. They may therefore also have the potential to adapt to new climates as they change, thereby displaying resilience. We have no information on how rapidly *I. ricinus* will be able to respond to climate change, but if the response is due to phenotypic plasticity, their response should occur within a generation; if the response is due to evolutionary divergence, numerous generations might be required depending on the genetic variance in sensitivity to temperature. Experiments testing individual ticks would reveal the level of phenotypic variation within populations; genetic studies could reveal intrapopulation genotypic variation, while analysis of factors such as fat content and infection with *B. burgdorferi* s.l. may contribute to individual differences in activity (Herrmann and Gern 2010, 2012).

### Experimental limitations

As with all laboratory experiments, there are limitations in terms of realistically replicating what might occur in nature. First, comparisons of the UK and French populations should be made with some caution because they were carried out at different times and locations, albeit under identical conditions using the same individual incubators, the same loggers, and the same observers.

Second, in order to minimize tick mortality before the end of each experiment, the experimental periods were as short as possible, meaning that only a restricted temperature range could be tested. We chose the cooler part of the range of temperatures over which *I. ricinus* nymphs quest because changes in the lower temperatures will directly affect the length of the *I. ricinus* questing season. It would be interesting to examine the whole climate envelope from each population (i.e., testing *I. ricinus* questing at higher temperatures), for which we might expect ticks from warm climates to be more tolerant of heat than ticks from cool climates.

Third, our experiments tested the effect of temperature only, by maintaining a constant high (above 90%) relative humidity. However, several studies have demonstrated the importance to questing of saturation deficit (the drying power of the air; a function of both temperature and relative humidity; e.g., Perret et al. 2000). For any given relative humidity, as the temperature increases, the saturation deficit will increase. Therefore, in theory, our results may be applicable primarily to high humidity situations, rather than dry conditions. However, tick questing data in Tagliapietra et al. (2011) imply that our results should be applicable to many natural situations over the same range of temperatures (6–15°C) that we used in our experiments, because these relatively cool temperatures were rarely associated with saturation deficits high enough to inhibit questing.

In conclusion, we found that questing behavior in response to temperature varies between *I. ricinus* populations in relation to thermal climate with a higher proportion of the population questing at lower temperatures if they are from cooler climates. This suggests *I. ricinus* have adapted to climate and may, therefore, have the potential to adapt to new climates as they change, thereby displaying resilience. Our findings have implications for predicting the distribution, activity, and disease risk posed by *I. ricinus* over different regions and under climate change.

## Supporting Information

Figure S1 The mean proportion of *Ixodes ricinus* nymphs questing for each count of the experiment for ticks from northeast Scotland (black squares), North Wales (open circles), South England (black triangles) and crosses (central France).
