# Supplementary material for: Climate of origin affects tick (Ixodes ricinus) host-seeking behavior in response to temperature: implications for resilience to climate change?
Source: Ecol Evol. 2014 Mar 10;4(7):1186–98. doi: 10.1002/ece3.1014 (PMC3997332; doi:10.1002/ece3.1014)
Supplement: Figure S1 — The mean proportion of Ixodes ricinus nymphs questing for each count of the experiment for ticks from northeast Scotland (black squares), North Wales (open circles), South England (black triangles) and crosses (central France). [file ece30004-1186-sd1.docx]

Supporting information, Gilbert et al.

Figure S1


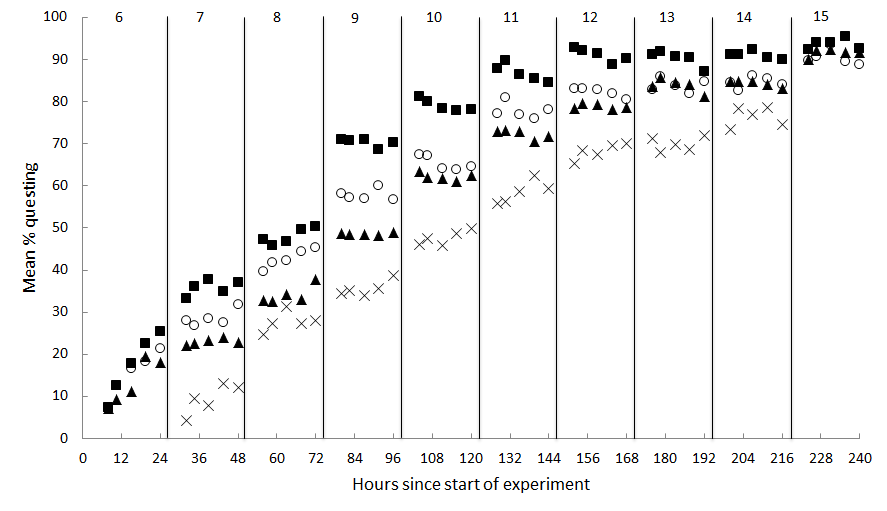


Fig. S1. The mean proportion of *I. ricinus* nymphs questing for each count of the experiment for ticks from NE. Scotland (black squares), N. Wales (open circles), S. England (black triangles) and crosses (central France). The vertical lines and numbers across the top of the figure indicate the experimental temperature in the incubators at the time of each count. Note that French ticks were not subject to temperatures of 6 or 15°C (and therefore the hours on the x-axis need to be subtracted by 24h to be correct for French ticks). Also note that all ticks were kept at 5°C for at least 2 hours before the start of each experiment.
